# *SORBS2* and *TLR3* induce premature senescence in primary human fibroblasts and keratinocytes

**DOI:** 10.1186/1471-2407-13-507

**Published:** 2013-10-29

**Authors:** Melanie Liesenfeld, Sandy Mosig, Harald Funke, Lars Jansen, Ingo B Runnebaum, Matthias Dürst, Claudia Backsch

**Affiliations:** 1 Department of Gynecology, Jena University Hospital, Friedrich Schiller University Jena, Bachstr. 18, Jena 07743, Germany; 2 AG Molecular Hemostaseology, Jena University Hospital, Friedrich Schiller University Jena, Bachstrasse 18, Jena 07743, Germany

**Keywords:** Human papillomavirus (HPV), Cervical carcinogenesis, Tumor suppressor genes, Primary keratinocytes, Lentiviral mediated gene transduction

## Abstract

**Background:**

Genetic aberrations are required for the progression of HPV-induced cervical precancers. A prerequisite for clonal expansion of cancer cells is unlimited proliferative capacity. In a cell culture model for cervical carcinogenesis loss of genes located on chromosome 4q35→qter and chromosome 10p14-p15 were found to be associated with escape from senescence. Moreover, by LOH and I-FISH analyses a higher frequency of allele loss of these regions was also observed in cervical carcinomas as compared to CIN3. The aim of this study was to identify candidate senescence-related genes located on chromosome 4q35→qter and chromosome 10p14-p15 which may contribute to clonal expansion at the transition of CIN3 to cancer.

**Methods:**

Microarray expression analyses were used to identify candidate genes down-regulated in cervical carcinomas as compared to CIN3. In order to relate these genes with the process of senescence their respective cDNAs were overexpressed in HPV16-immortalized keratinocytes as well as in primary human fibroblasts and keratinocytes using lentivirus mediated gene transduction.

**Results:**

Overall fifteen genes located on chromosome 4q35→qter and chromosome 10p14-p15 were identified. Ten of these genes could be validated in biopsies by RT-PCR. Of interest is the novel finding that SORBS2 and TLR3 can induce senescence in primary human fibroblasts and keratinocytes but not in HPV-immortalized cell lines. Intriguingly, the endogenous expression of both genes increases during finite passaging of primary keratinocytes in vitro.

**Conclusions:**

The relevance of the genes SORBS2 and TLR3 in the process of cellular senescence warrants further investigation. In ongoing experiments we are investigating whether this increase in gene expression is also characteristic of replicative senescence.

## Background

One of the inherent properties of high-risk human papillomavirus (HR-HPV) types belonging to the α-genus of the *Papillomaviridae* is their ability to immortalize primary human epithelial cells. In pioneering experiments conducted in the late eighties it was shown that transfection of the native HPV16 or HPV18 genomes could extend the proliferative life-span of foreskin keratinocyte cultures. After a phase of crisis, immortal cell clones would emerge from most of these cultures [1,2]. In subsequent experiments the viral proteins driving the immortalization process were identified [3,4]. The oncoproteins E6 and E7 interact with numerous cell regulatory proteins, most notably with p53 and pRb respectively, thereby deregulating the cell cycle, DNA repair and apoptosis which inevitably leads to genetic instability [5,6]. Indeed, the inactivation of the p16^INK4A^-Rb pathway has more recently been identified to play a critical role in cellular senescence [7,8]. However, bypassing one of the regulatory pathways for senescence is not sufficient for immortal cell growth. Several additional genetic alterations including the activation of telomerase are indispensable for immortality [9]. Somatic cell fusion experiments suggest that the immortal phenotype can be complemented and thereby implies loss of gene function as a characteristic step in this process [10,11]. In our previous studies using microcell-mediated chromosome transfer into HPV-immortalized cells we provided functional evidence for senescence associated genes located on chromosomal regions 10p14-15 and 4q35→qter [12,13]. Importantly, these regions are more frequently affected by allele loss in cervical carcinomas than precancers [12-14] and thus warranted a more detailed investigation. In order to identify candidate genes which contribute to senescence we had in earlier experiments performed microarray expression analyses with a particular focus on the above chromosomal regions. Two genes were found to be significantly down-regulated in cervical cancers as compared to precancers (CIN3). However, ectopic expression of neither gene could induce senescence in HPV-transformed cells [15]. A possible weakness of that study may have been the use of pooled RNA for the array experiments which may have masked differences in the expression levels of several genes. In the present study we have therefore modified our strategy. Instead of pooled RNA, RNA from 12 microdissected CIN3 and 11 cervical carcinomas were used for individual expression analyses. Ten out of 15 genes which were found to be significantly down-regulated in cervical cancers versus CIN3 could be validated by quantitative RT-PCR and showed highly significant differences in expression between the two groups. Of seven genes cDNAs were overexpressed in HPV-immortalized cell lines, primary fibroblasts or primary keratinocytes to test their potential to induce senescence.

## Methods

### Tissue specimens

Biopsies of histopathologically confirmed CIN3 and squamous cell carcinoma (CxCa) from patients at the Department of Gynaecology at the Jena University Hospital were available for this study. This study was approved by the Ethics Committee of the Friedrich-Schiller-University Jena (reference number: 1402-09/04). Informed consent for the use of residual tissue for research was given. All cases were HPV-16 positive. Biopsies were embedded in Tissue-Tek (Sakura Finetek Germany GmbH, Staufen, Germany) and stored at -80°C. Sectioning was performed using a cryo-microtome (Shandon cryotome SME, Thermo Fisher Scientific, Wilmington, USA). Sections of 12–15 μm thickness were prepared. To protect the RNA from degradation, all instruments used for RNA isolation were treated with RNaseZap (Ambion/Applied Biosystems, Darmstadt, Germany) and rinsed with DEPC-water. Sections were placed on pre-cooled PALM MembraneSlides NF (RNase- and DNase-free; PALM Microlaser Technology, Bernried, Germany) which were treated with UV-light for 30 min thereby allowing better adhesion. The slides were quickly dried on a thermoplate at 40°C to protect tissue and RNA from humidity. Tissue-Tek medium interferes with the laser beam during microdissection and was therefore removed by washing the slides in 70% ethanol followed by drying on a thermoplate.

### Laser capture microdissection

Immediately before microdissection the tissue sections were stained with a 1%-solution of Cresyl Violet acetate (minimum dye content 70%, Sigma-Aldrich, Seelze, Germany) in 96% ethanol for 1 min, differentiated with 70% ethanol and dried on a heating plate. Microdissection and pressure catapulting of tumor or dysplastic areas were performed on a PALM MicroBeam (Zeiss, Jena, Germany). The specimens were collected in PALM AdhesiveCaps 500 opaque (PALM Microlaser Technologies, Bernried, Germany).

### RNA isolation, RNA quality and integrity assessment

The microdissected samples were dissolved in 400-500 μl TRIzol reagent (Invitrogen, Karlsruhe, Germany) and mixed with 0.33 volumes of chloroform. For a clear separation of the aqueous and organic phase the TRIzol-Chloroform solution was centrifuged at 14000 rpm for 20 min at 4°C in Phase Lock Gel Heavy tubes (Eppendorf, Hamburg, Germany). The aqueous phase was collected and mixed with 2 volumes of 70% ethanol. Total RNA was extracted with the RNeasy Micro Kit (QIAGEN, Hilden, Germany) according to the manufacturer’s instructions (which include DNase digestion) and was eluted in 14 μl RNase-free water. To assess the concentration of the total RNA, 1 μl was directly measured on a NanoDrop spectrophotometer (ND-1000, NanoDrop Technologies, Thermo Fisher Scientific, Wilmington, USA). The integrity of the RNA was determined using a 2100 Bioanalyzer (Agilent Technologies, Waldbronn, Germany) thus enabling quality comparison to be made between the samples. The median RIN (RNA integrity number) of the RNA samples, which were hybridised to microarrays, was 6.2 (+/- 1.12).

### Microarray hybridization and data analysis

Finally, RNA from 12 microdissected CIN3 and 11 CxCa were used for Agilent Whole Human Genome Oligo Microarray (4 × 44 K slide) hybridization in one-colour experiments with Cy3-labeled samples. Reverse transcription into cDNA, labeling of cDNA, hybridization and scanning of the microarrays were done according to the manufacturers’ protocols. Quality control was performed with the Feature Extraction Software by Agilent. Background signals were eliminated and all gene expression values were normalized to the median. Genes with expression values over 20 were set as expressed. Further gene analyses were performed with the software GeneSpring from Agilent. For group comparison the Welch t-test with a p-value cut-off of 0.05 was applied, followed by a multiple testing correction according to Benjamini and Hochberg [16].

### Validation with real-time PCR (qPCR)

The microarray data of all candidate genes was validated by reverse transcription qPCR. For this purpose the remaining RNA from the array analyses and from further microdissected biopsies, altogether 20 CIN3 and 20 CxCa, were used. All cases were HPV16-positive.

200 ng RNA of each sample was reverse transcribed in a total volume of 40 μl using SuperScript II Reverse Transcriptase (Invitrogen, Karlsruhe, Germany) according to the manufacturers’ protocol. Quantitative PCR was done with an ABI 7300 SDS system (Applied Biosystems, Darmstadt, Germany) using the PowerSybrGreen Master Mix (Applied Biosystems, Darmstadt, Germany). Reactions were performed with cDNA equivalent to 5 ng RNA in a volume of 25 μl volume comprising gene-specific forward and reverse primers (10pmol each) (Additional file 1: Table S1). To determine the normalisation factor (NF) we used the three most stable housekeeping genes (HKGs) *GAPDH*, *HPRT* and *ACTB*, which were identified by the method of Vandesompele and colleagues [17]. PCR data were analysed using REST (relative expression software tool) [18].

### Amplification, cloning and sequencing of cDNA

To generate expression-constructs of the genes of interest we used cDNA-clones available from ImaGenes (Berlin, Germany) (Additional file 2: Table S2). The coding sequences were amplified with the Long Expand Template PCR-System (Roche, Mannheim, Germany). The PCR comprised dNTP’s (350 μM), forward and reverse primers (300 nM), 1× buffer, plasmid-DNA (10 ng), enzyme-mix (3.75 U). The following PCR cycling parameters were employed: 94°C for 2 min initial denaturation, followed by 10 cycles at 94°C for 10 s, annealing temperature according to Table S3 (Additional file 3: Table S3) for 30 s, elongation at 68°C for 2 min. The next 20 cycles consisted of 94°C for 15 s, 65°C for 30 s, 68°C for 2 min with an additional increase of 20 s at 68°C for every cycle. The PCR products were separated on 2% agarose gels and the bands of predicted size were excised. DNA was eluted with Zymoclean Gel DNA Recovery Kit (Zymo research, Orange, USA) following the manufacturers protocol. PCR-products were cloned into pJet using the CloneJET PCR Cloning Kit (Fermentas, St. Leon-Rot, Germany) following the manufacturer’s instructions and transformed into E.coli XL1-Blue. Subcloning into the lentiviral vector pCDH-CMV-MCS-EF1-Puro (#CD510B-1, BioCat, Heidelberg, Germany) was done via the restriction enzymes shown in Table S4 (Additional file 4: Table S4). The ligation reaction consisted of: 50 ng pCDH-vector; 3U Ligase (Fermentas, St. Leon-Rot, Germany); 2 μl 10× buffer and 10 ng DNA-fragment in a total volume of 20 μl. The mix was incubated at 14°C for 16 h and then cloned into E.coli Stbl3 (Invitrogen, Karlsruhe, Germany). To ensure functionality all constructs were sequenced at Seqlab (Göttingen, Germany) (Additional file 5: Table S5).

### Cell culture

For functional studies, the following cell lines were used: HPKIA and HPKII (both HPV16-immortalized human keratinocytes at passages p83 and p289, respectively) [19,20], CaSki and SiHa (both HPV16-positive cervical carcinoma), HeLa, SW756 and C4.I (all HPV18-positive cervical carcinoma). All cervical carcinoma cell lines were obtained from the ATCC. Primary human keratinocytes and fibroblasts were isolated from foreskins of four donors. Primary keratinocytes were cultured in EpiLife (Cascade Biologics, Portland, USA) with human keratinocyte growth supplement (HKGS, Cascade Biologics, Portland, USA). All other cell lines were grown in Dulbecco’s modified Eagle medium (D-MEM; Gibco Invitrogen, Karlsruhe, Germany) supplemented with 10% fetal calf serum (FCS), 100 U/ml penicillin and 100 μg/ml streptomycin at 37°C with 5% CO_2_.

### Lentivirus production

For the generation of recombinant lentivirus particles HEK 293 T cells were transfected using the calcium phosphate-method. One day before transfection 1.8×10^6^ cells were seeded into a 6 cm-plate to achieve 80-90% confluence by the next day. The transfection mix comprising CaCl_2_ and BES-buffered saline, 2 μg pCDH expression plasmid, 2 μg pMDL, 1 μg pRSV and 0.4 μg pVSV-g was dropped onto the cells into 3 ml D-MEM (+10% FCS) and cultured over night at 35°C and in 3%CO_2_. The next day the cells were washed 2× with PBS and cultured over night with 3 ml medium at 32°C and in 5% CO_2_. About 48 h after transfection the virus-containing medium could be harvested. A 3rd generation packaging system with plasmid pMDLg/pRRE (included HIV-1 gag/pol genes; gag, coding for the virion main structural proteins; pol, responsible for the retrovirus-specific enzymes; RRE, a binding site for the Rev protein), plasmid pRSV-Rev (included parts of HIV-1 rev gene, controlling the export rate of mRNAs) and plasmid pCMV-VSVG (envelope plasmid) were used to produce lentiviral particles.

### Lentiviral gene transduction

One day before transduction the target cells were seeded into 12-well-plates at a density of 1×10^6^ cells/well. Virus-containing medium was harvested and filtered through a 0.45 μm filter. For each well 1 ml of virus-containing medium was mixed with 2 μg Polybrene (stock 5 mg/ml; Sigma-Aldrich, Steinheim, Germany) to replace the culture medium. Transduction was performed twice on the first day, temporally separated by 6 h, and a third time on the next day. Polybrene was used only for the first transduction. After adding the virus-containing medium the plates were centrifuged at 1500 rpm for 45 min at RT and subsequently cultured at 32°C and in 5% CO_2_ in the incubator. Six hours after the third transduction the virus-containing medium was removed and replaced with unmodified culture medium and the cells were cultured under normal conditions. For establishing stable clones selection was performed with 0.5 μg - 1 μg/ml puromycin hydrochlorid (Sigma-Aldrich, Steinheim, Germany) for 5–7 days. Assays were done in triplicate and repeated four times.

### β-galactosidase assay

After removing the culture medium, cells were washed in PBS and fixed in a 3% formaldehyde solution for 5 min at RT. Cells were washed again with PBS and incubated with fresh senescence-associated β-galactosidase (SA-β-gal) solution as described by Dimri and colleagues [21]. Incubation was performed at 37°C over night.

### Northern blot

Northern blotting was done according to a standard protocol. Approximately 4 μg of total RNA were separated in a 1% MOPS- agarose gel and blotted onto a nylon membrane. Radioactively labeled DNA probes were generated by random priming (Roche Applied Science, Mannheim, Germany) with [^32^P]-ATP (Hartmann Analytic GmbH, Braunschweig, Germany). For removal of non-integrated nucleotides G-50 Sephadex-columns (Roche Applied Science, Mannheim, Germany) were used. Hybridization was performed for 2–3 days at 42°C followed by stringent washing and exposition to X-ray films for 3-14d at -80°C.

### Immunofluorescence

1×10^5^ cells suspended in 150 μl PBS were centrifuged at 500 rpm for 2 min at RT onto a glass slides. After fixation with 4% paraformaldehyde for 10 min, slides were washed with Tris-buffered solution (50 mM Tris, 150 mM NaCL) with 0,1% Tween-20 (TBST). Slides were then blocked with 50 μl normal serum (donkey-serum for SORBS2- and goat-serum for p16^INK4a^- antibody, respectively)(each 1:5 dilution in TBST) for 20 min at RT. Primary antibodies specific for SORBS2 (ArgBP2 H-15, Santa Cruz Biotechnologies, Heidelberg, Germany) (dilution 1:200) or p16^INK4A^ (Roche mtm Laboratories, Heidelberg, Germany) (dilution 1:2) were then applied and incubated over night at 4°C followed by 3 washing steps with 1× TBST for 5 min each. Incubation with the secondary antibody for SORBS2 staining (donkey anti-goat IgG-FITC sc-2024, 1:100; Santa Cruz Biotechnologies, Heidelberg, Germany) was done for 45 min at RT. Afterwards slides were washed again 3 times for 5 min with washing buffer, rinsed with aqua bidest and covered with antifade/DAPI-solution under a coverslip. P16^INK4a^ -detection was performed using EnVision™Detection System Rabbit/Mouse (Dako Deutschland GmbH, Hamburg, Germany) according to the manufacturers protocol. Slides were then counterstained with hematoxylin and covered with coverslips in gelatine. Fluorescence or brightfield images were obtained with a Zeiss Axioplan 2 microscope and a Axio Cam HRc using 20× and 40× objectives using AxioVision Application Rel.4.5.2, (Zeiss, Jena, Germany).

### Western blot

Western blotting was done according to a standard protocol for semi dry procedure. Cells growing in a 6 cm dish were lysed in 300 μl of 1% SDS solution with 1:100 protein inhibitor (Serva Electrophoresis, Heidelberg, Germany). Approximately 10-15 μg protein was separated by 10% SDS-PAGE and transferred to an Immobilon-P membrane (Millipore, Bedford, USA). For immunostaining antibodies specific for SORBS2 (ArgBP2 H-15 goat, 100 ng/ml, Santa Cruz Biotechnologies, Heidelberg, Germany), TLR3 (TLR3 L-13, N-14 and Q-18, each goat IgG, 200 ng/ml, Santa Cruz Biotechnologies, Heidelberg, Germany) and ACTB (ACTB mouse, 125 ng/ml, BD Bioscience, Heidelberg, Germany) were used. The secondary antibodies were labelled with horseradish peroxidase (HRP) (anti-rabbit 0.04 ng/ml, anti-goat 0.08 ng/ml, anti-mouse 0.04 ng/ml; Dianova, Hamburg, Germany). The first antibodies were incubated over night at 4°C and the second antibodies for 1 h at RT. A 5% milk solution was used for blocking. All antibodies were diluted in 1% BSA solution. Staining was visualized using enhanced chemiluminescence (SuperSignal West Pico Chemiluminescent Substrate, Thermo Fisher Scientific, Dreieich, Germany).

## Results

### Identification of senescence-associated genes by microarray analyses

For microdissection only biopsies with large cohering tumor areas, a low amount of lymphocyte infiltrates and sharp-cut borderlines to stroma were chosen. Overall 35 CIN3 and 64 CxCa were microdissected and RNA was isolated. Of these 12 CIN3 and 11 CxCa yielded RNA with an integrity number (RIN) of ≥5 and a concentration of 25 ng/μl which were considered to be the minimal required criteria for microarray analyses. Evaluation of the microarrays primarily focussed on the chromosomal regions 4q35→qter and 10p14-15 for which we have functional evidence for the location of tumour suppressor genes [12,13]. Because only 85 (4q35→qter) and 95 (10p14-15) genes are located within these regions the threshold level indicative for differences in gene expression was lowered to 1.3 which is below the standard value of 2. Overall 15 genes were found to be significantly down-regulated in cervical cancers versus CIN3 (Table 1).

**Table 1:** Down-regulated genes located on 4q35 and 10p14-15

**Agilent-Nr**	**Gene**	**Symbol**	**Chromosome**	**Expression Array**	**Real-time PCR**
				**Ratio CIN3/CxCa (p-value)**	
A_23_P104413	double homeobox, 4	*DUX4*	4q35	-1.92 (0.0382)	n.d.
A_23_P121795	sorbin and SH3 domain containing 2	*SORBS2*	4q35.1	-1.83 (0.0487)	-31.709 (<0.001)
A_23_P140884	tubulin, beta polypeptide 4, member Q	*TUBB4Q*	4q35	-1.76 (0.0409)	n.d.
A_23_P29922	toll-like receptor 3	*TLR3*	4q35	-2.3 (0.0024)	-6.437 (<0.001)
A_23_P358470	coiled-coil domain containing 111	*CCDC111*	4q35.1	-1.56 (0,0213)	n.d.
A_23_P58180	cytochrome P450, family 4, subfamily V, polypeptide 2	*CYP4V2*	4q35.2	-1.96 (0.0446)	-6.144 (<0.001)
A_23_P104252	inter-alpha (globulin) inhibitor H5	*ITIH5*	10p14	-4.14 (0.0301)	n.d.
A_23_P12849	F-box protein, helicase, 18	*FBXO18*	10p15.1	-1.34 (0.0228)	-2.445 (<0.001)
A_23_P1374	protein kinase C, theta	*PRKCQ*	10p15	-3.69 (0.0428)	-8.048 (<0.001)
A_23_P138680	Interleukin 15 receptor, alpha	*IL15RA*	10p15-p14	-1.68 (0.00312)	-2.973 (<0.001)
A_23_P47073	WD repeat domain 37	*WDR37*	10p15.3	-1.39 (0.0355)	-4.676 (<0.001)
A_23_P75056	GATA binding protein 3	*GATA3*	10p15	-1.5 (0.0222)	-30.061 (<0.001)
A_24_P111096	6-phospho-fructo-2-kinase /fructose-2,6-bisphosphatase	*PFKFB3*	10p14-p15	-1.69 (0.017)	-1.971 (0.0174)
A_24_P242705	tRNA aspartic acid methyl-transferase 1	*TRDMT1*	10p15.1	-1.55 (0.0238)	n.d.
A_24_P360722	DIP2 disco-interacting protein 2 homolog C (Drosophila)	*DIP2C*	10p15.3	-1.39 (0.0433)	-3.368 (<0.001)

n.d. – not determined.

### Validation of senescence-associated genes by RT-PCR

**Figure 1:**
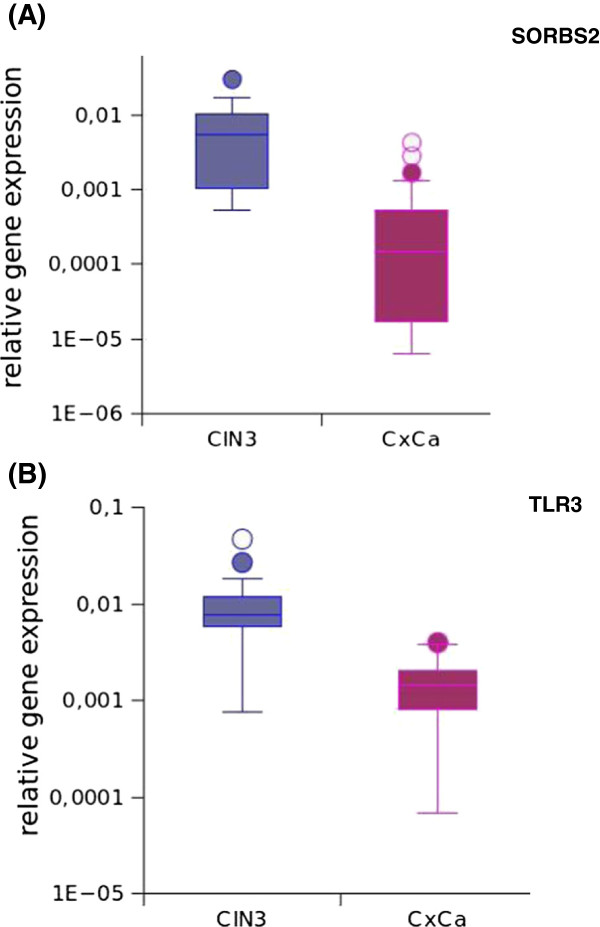
**SORBS2 and TLR3 expression in CIN3 and cervical carcinomas (CxCa).** Relative gene expression of SORBS2 **(A)** and TLR3 **(B)** was determined by quantitative RT-PCR in CIN3 (n = 20) and CxCa (n = 20). For normalisation the housekeeping genes GAPDH, HPRT and ACTB were used. Both genes were expressed at highly significant (p < 0.001) lower levels in CxCa than in CIN3 **(A**, **B)**.

Ten genes could be validated by quantitative RT-PCR and showed highly significant differences in expression between the two groups (Table 1). The validation set comprised not only RNA of the biopsies used for array analyses but also an additional set of 8 CIN3 and 9 CxCa. Of note is, that with exception of PFKFB3 the differences in gene expression remained highly significant even when evaluation was based solely on the independent set of RNAs (Additional file 6: Table S6). For five of the 15 genes reliable RT-PCR assays could not be established and were therefore not pursued further at this stage. Relative gene expression levels of SORBS2 and TLR3 in CIN3 and CxCa are shown in Figure 1 and for all other genes in Figure S1 (Additional file 7: Figure S1).

### Ectopic expression of candidate genes

The effect of ectopic expression of *SORBS2*, *TLR3*, *CYP4V2*, *FBXO18*, *IL15RA*, *WDR37* and *DIP2C* on the cell phenotype, in particular senescence, was investigated in primary cells, HPV-immortalized cells and cervical carcinoma cells. *PFKFB3* was not pursued further because down-regulation could not be validated in an independent set of biopsies (Additional file 6: Table S6).

All cDNAs were cloned into the pCDH vector for lentivirus mediated gene transduction (Additional file 2: Table S2). For two candidate genes, *PRKCQ* and *GATA3* the cDNA clones were erroneous. The empty vector served as negative control in all experiments. Two different splice variants were used for *SORBS2*. Both splice variants (SORBS2-1 and SORBS2-2) code for the SoHo domain at the N-terminus and the three major SH3-domains at their COOH-terminal region.

**Figure 2:**
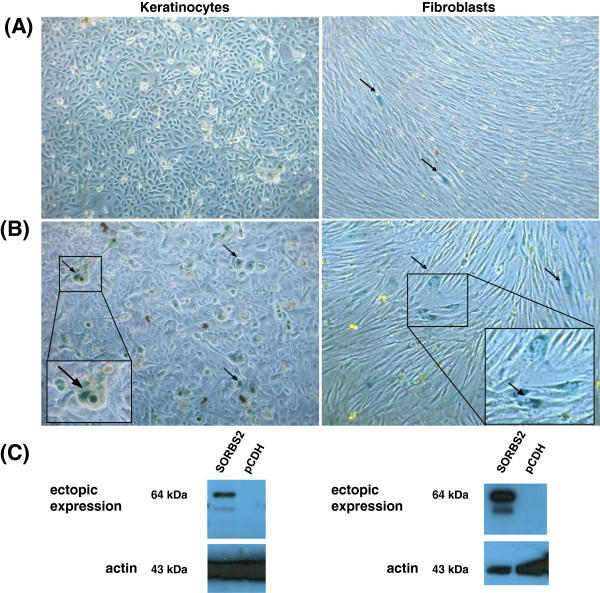
**Induction of senescence after ectopic expression of SORBS2 in primary fibroblasts and keratinocytes.** Beta-galactosidase staining was performed at different time points after lentiviral transduction. Depicted are cells stained 14 days after transduction of pCDH empty vector (control) and SORBS2-2. Only a few senescent cells (arrows) are evident in the control **(A)**. An at least 4-fold increase in senescent cells is observed after transduction of SORBS2 both in primary human fibroblasts and keratinocytes **(B)**. All images were captured at 200× magnification. Expression of the transgene in cells at same passage was confirmed by Western blot **(C)**.

All candidate genes were ectopically expressed in primary keratinocytes and fibroblasts and in the HPV16-immortalized keratinocyte cell lines HPKIA p83 and HPKII p289. Overall these two cell lines showed the lowest endogenous expression of the genes to be analysed (Table 2). Selection was done with puromycin. Stable expression over at least five passages was confirmed by Northern blots and in case of SORBS2 also by Western blot (Figure 2, Additional file 8: Figure S2 and Additional file 9: Figure S3).

**Table 2:** Relative expression of candidate genes in different cell lines

**Gene**	**SORBS2**	**TLR3**	**CYP4V2**	**FBXO18**	**IL15RA**	**WDR37**	**DIP2C**
**cell line**							
HPKIAp83	***-125301***	-76	-400	-62	-97	-80	***-1006***
HPKIAp359	-19415	-48	-316	-48	-37	-47	-178
HPKIIp54	-30612	-223	-905	***-134***	***-129***	-149	***-509***
HPKIIp289	-51328	***-504***	***-1560***	***-120***	***-430***	-122	-457
CaSki	-678	-190	***-1603***	-97	-85	***-158***	-409
SiHa	***-56208***	***-515***	-872	-33	-55	-71	-139
HeLa	-345	-248	-631	-20	-13	-66	-348
SW756	-3674	-234	-1299	-56	-35	***-154***	-165
C4.1	-1881	-35	-364	-46	-36	-47	-49

Calculation is based on the formula: -1/2^(Ct mean housekeeping genes – Ct gene of interest)^.

The lowest expression values are shown in bold.

#### ***None of the genes could induce senescence in HPV-immortalized cells and cervical carcinoma derived cell lines***

All seven candidate genes were ectopically expressed in HPKIA p83 and HPKII p289 cells. In addition, their influence in SiHa cells and SW756 cells was also examined. Three days post transduction the cells were seeded in 6 well plates to be stained by the β-galactosidase assay at different time points up to 3 weeks. Staining was performed whenever the cultures reached 80-90% confluence. To monitor induction of senescence over this time span the cells needed to be sub-cultured whenever confluence was reached. No differences in cell growth were noted when cells transduced with the gene of interest and the empty vector (pCDH) were compared. All transductants were selected for puromycin resistance and were passaged beyond 6 weeks before being frozen for storage. Senescence above background was not observed in any of the experiments conducted (Additional file 8: Figure S2).

#### ***SORBS2 and TLR3 could induce senescence in primary fibroblasts and keratinocytes***

We then decided to investigate whether the candidate genes could induce senescence in primary human foreskin fibroblasts and keratinocytes. Low passage cells (p2) were transduced in the same manner as immortal cell lines. The highest number of senescent cells was observed for SORBS2-2 fourteen days after transduction. Thereafter, the number of senescent cells decreased most likely due to the lack of detachment or reattachment of senescing cells during sub-culturing. The characteristic perinuclear β-galactosidase staining pattern of enlarged and flattened cells was observed in case of fibroblasts (Figure 2). In contrast, the morphology of senescing keratinocytes differed in that both enlarged and rounded cells with an overall blue stain were observed (Figure 2). Senescence was scored positive if the number of β-galactosidase stained cells was ≥ 2-fold (+) or ≥ 4-fold (++) higher as compared to empty vector. Altogether fibroblasts were more responsive for senescence than keratinocytes (Table 3). SORBS2-2 and TLR3 could induce senescence in both cell types whereas for SORBS2-1 the effect was restricted to fibroblasts. Moreover, CYP4V2 and FBXO18 could induce senescence in fibroblasts only but the results of four independent experiments were inconsistent.

**Table 3:** Summary of the investigated genes and their influence on senescence in primary fibroblasts and keratinocytes (4 independent experiments each)

**Gene**	**Fibroblasts**	**Keratinocytes**
*SORBS2-1*	+	-
*SORBS2-2*	++	++
*TLR3*	+	+
*CYP4V2*	+ / -	-
*FBXO18*	+ / -	-
*IL15RA*	-	-
*WDR37*	-	-
*DIP2C*	-	-

-no differences between expression of the gene and pCDH (empty vector).

+ / -≥ 2-fold number of senescent cells in comparison to the empty vector, but inconsistent between experiments.

+≥ 2-fold number of senescent cells in comparison to the empty vector.

++≥ 4-fold number of senescent cells in comparison to the empty vector.

### Endogenous levels of SORBS2 and TLR3 increase during in vitro passaging of primary keratinocytes

**Figure 3:**
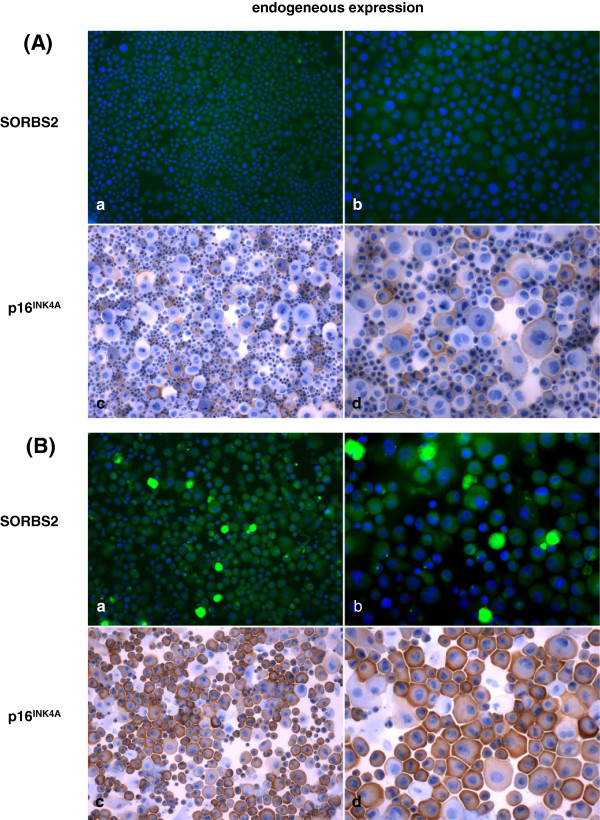
**Endogenous expression of SORBS2 and p16**^**INK4A **^**during in vitro passaging of primary keratinocytes.** Immunocytochemical staining of endogenously expressed SORBS2 and p16^INK4A^ in primary human keratinocytes of the donor FK09-9 **(A)** at passage 5 and **(B)** at passage 7 was performed. Strong endogenous expression of p16^INK4a^ and SORBS2 correlated with a near senescent stage of the cells **(B)**. (a, c magnification ×100; b, d magnification ×200)

The life span of primary keratinocytes in culture is short. Normally the cells reach a phase of crisis within 10 passages. This is in sharp contrast to fibroblasts which can easily be passaged 50 times and more before entering crisis. Since SORBS2-2 and TLR3 can induce senescence after ectopic expression in primary keratinocytes we were curious to evaluate the endogenous expression of these genes during finite passaging of keratinocytes. Expression levels were determined by quantitative RT-PCR and in case of SORBS2 also by immunocytochemistry. Increased p16^INK4a^ expression, a hallmark of senescing cells, was monitored in parallel. Primary cultures at the earliest available passage (p2) expressed very low relative levels of SORBS2 and TLR3 which could be detected by qRT-PCR but not by Northern blots or immunocytochemistry (data not shown). In subsequent passages a strong increase in endogenous expression of SORBS2 and TLR3 was observed in 3 primary keratinocytes cultures from different donors. Exemplarily, immunostaining for SORBS2 and p16^INK4a^ and qRT-PCR for TLR3 at passage 5 and 7 are shown in Figures 3 and in Additional file 10: Figure S4, respectively. Strong endogenous expression of p16^INK4a^, SORBS2 and TLR3 correlated with a near senescent stage of the cells.

## Discussion

Escape from senescence is a prerequisite for carcinogenesis. Our previous functional studies involving somatic cell fusions and micro-cell mediated chromosome transfers have linked recessive gene alterations with the acquisition of immortality [12,13,22]. In this study we have identified and validated 10 genes located within the chromosomal regions 4q35 → qter and 10p14-p15 that were down-regulated in cervical cancer as compared to CIN3. Down-regulation of SORBS2, TLR3 and GATA-3 has already been described in the context of cervical carcinogenesis. SORBS2 was shown to be expressed in normal cervical epithelium and CIN3 but at significantly reduced levels in CxCa [15]. Immunhistochemical analysis for GATA-3 showed a clear nuclear staining in normal cervical squamous epithelium, CIN1 and CIN2 lesions, but 11% of CIN3 and 67% of CxCa revealed a complete absence of GATA-3 immunostaining [23]. TLR3, a toll-like receptor eliciting an antiviral response in the presence of HPV oncogene expression, is increased in CIN3 followed by a decrease in CxCa [24]. The remaining 7 genes with exception of PFKFB3 are not linked to cancer when reviewing the literature. PFKFB3, in particular the splice variant UBI2K4 is considered to be a tumor suppressor protein in glioblastomas [25].

None of the above genes have thus far been associated with the loss of senescence. We had expected that reconstitution of gene expression in HPV-immortalised keratinocyte cell lines such as HPKIA and HPKII or cervical carcinoma derived cell lines would induced senescence. However, this was not the case and leaves room for speculation. In previous analyses using micro-cell mediated transfer either of the entire chromosome 4 or 10 resulted in senescence of HPKII cells. In subsequent studies using derivative of chromosomes 4 and 10 the chromosomal region harbouring putative senescence genes were narrowed down by exclusion [12,13]. In other words none of the derivative chromosomes could induce senescence. It is therefore conceivable that a candidate senescence gene is indeed located within this region but to exert its effect on senescence the product of another gene located on the same chromosome is required. If this gene is lacking, ectopic expression of the senescence gene itself will have no effect. We have therefore ectopically expressed the candidate genes in primary human keratinocytes and fibroblasts which should not have any genetic aberrations. Of the seven candidate genes only SORBS2-2 and TLR3 could reproducibly induce senescence both in fibroblasts and keratinocytes. SORBS2-1, a shorter splice variant of SORBS2, could only induce senescence in fibroblasts. The reason for this phenomenon is unclear. Both isoforms encode relevant functional parts of the protein such as the SoHo (Sorbin homology) and the three SH3 (Src homology 3) domains at the amino and carboxyl terminal end, respectively.

Clearly, the type of senescence observed in this study is not to be confused with proliferative senescence which results when telomeres have reached a critical minimal length and thereby have lost their protective structure. Senescence can also be induced in the absence of telomere shortening or dysfunction by a variety of conditions. This phenomenon is referred to as premature senescence and is either induced by stress as a consequence of suboptimal culturing conditions [26], or by elevated oncogene expression [9]. Indeed, aberrant activation of signal transduction pathways and promoting cell cycle regulators can result in a DNA-damage response (DDR) and thereby induce premature senescence. In this context the role of c-Abl in DNA repair may be helpful to understand our finding that SORBS2 can induce senescence. SORBS2 interacts with poly-proline motifs of c-Arg and c-Abl kinases via its three SH3 domains. A complex of the ubiquitin ligase Cbl and SORBS2 mediates the ubiquitination and degradation of c-Abl [27]. Within this complex, SORBS2 acts as a regulator of ubiquitination and degradation of c-Abl. It remains to be shown if this pathway is relevant for premature senescence. At a first glance the association between TLR3 and senescence may be interpreted as a lentivirus induced phenomenon. TLR3 is a member of the toll-like receptor family involved in the response of the innate immune system to pathogens such as double-stranded RNA viruses [28]. TLR3 activation results in the induction of intracellular pathways mediated by NF-қB and MAPK and can lead to IFN-beta and TNF-alpha gene expression [29,30]. Furthermore, NF-қB activation can lead to the production of proinflammatory cytokines expression such as IL-1 and apoptosis [28] or IL-1 and IFN-beta induced senescence [31,32]. We can therefore not completely exclude that the senescence observed in the TLR3 encoding lentivirus infected primary cells reflects a bias of the expression system. However, it should be considered that the very same recombinant virus could not induce senescence in HPV16-immortalized HPK cells. Moreover, the “empty” virus which served as negative control in all experiments, could not induced senescence above background in any of the cell lines or primary fibroblasts or keratinocytes analysed.

The observation that the endogenous expression of both SORBS2 and TLR3 is increased drastically in primary keratinocyte cultures at near senescent passage provides further support that these genes may contribute to senescence. In ongoing experiments we are investigating whether this increase in gene expression is also characteristic of replicative senescence. Moreover, modulation of both genes by shRNA-knockdown may result in a prolonged life-span of keratinocytes in culture which would be another indication for a physiological role of either protein in the senescence process.

## Conclusions

Of interest is the novel finding that SORBS2 and TLR3 can induce senescence in primary human fibroblasts and keratinocytes but not in HPV-immortalized cell lines. Intriguingly, the endogenous expression of both genes increases during finite passaging of primary keratinocytes in vitro. The relevance of these genes in the process of cellular senescence warrants further investigation.

## Competing interests

The authors declare that they have no competing interests.

## Authors’ contributions

ML performed and analysed experiments and wrote the manuscript, LJ contributed to the experiments, SM performed microarray experiments, HF supervised the microarray experiments, IBR supervised the study, MD and CB designed and supervised the study and revised the manuscript. All authors read and approved the final manuscript.

## Pre-publication history

The pre-publication history for this paper can be accessed here:

http://www.biomedcentral.com/1471-2407/13/507/prepub

## Supplementary Material

Additional file 1: Table S1 Primer for real-time PCR. Click here for file

Additional file 2: Table S2 Full-length cDNA-clones and source. Click here for file

Additional file 3: Table S3 PCR-terms for long expand template PCR for the different genes. Click here for file

Additional file 4: Table S4 Primers with restriction sites for long expand template PCR. Click here for file

Additional file 5: Table S5 Primers for sequencing of cDNA-clones. Click here for file

Additional file 6: Table S6 Validation with quantitative real-time PCR and evaluation with REST [18] CIN3 vs. CxCa. Click here for file

Additional file 7: Figure S1 Expression of candidate genes in CIN3 and cervical carcinomas (CxCa). Relative gene expression of WDR37, PFKFB3, IL15RA, FBXO18, DIP2, CYPV2, PRKCQ, GATA3 was determined by quantitative RT-PCR in CIN3 (n = 20) and CxCa (n = 20). For normalisation the housekeeping genes GAPDH, HPRT and ACTB were used. With exception of PFKFB3 the differences in gene expression were highly significant (<0.001). Click here for file

Additional file 8: Figure S2 Ectopic expression of SORBS2 in HPV-immortalized cells. Beta-galactosidase staining was performed at different time points after lentiviral transduction. Depicted are cells stained 14 days after transduction of pCDH empty vector (control) and SORBS2-2. Senescence could not be observed in HPKIAp83 and HPKIIp289 (B). Controls were also negative (A). All images were captured at 200× magnification. Expression of the transgene in cells at same passage was confirmed by Western blot (C). Click here for file

Additional file 9: Figure S3 Ectopic expression of TLR3 in primary fibroblasts Beta-galactosidase staining was performed at different time points after lentiviral transduction. Depicted are cells stained 14 days after transduction of pCDH empty vector (control) and TLR3. Only a few senescent cells are evident in the control (A). An at least 2-fold increase in senescent cells was observed after transduction of TLR3 in primary human fibroblasts (B). The images were captured at 200× magnification. Expression of the transgene in cells at passage 1, 3 and 5 was confirmed by Northern blot (C). Click here for file

Additional file 10: Figure S4 Endogenous expression of TLR3 during in vitro passaging of primary keratinocytes. Relative gene expression of TLR3 in two different passages from three different donors (FK09-3 (A), FK09-7 (B) and FK09-9 (C)) was determined by quantitative RT-PCR. For normalisation the housekeeping genes GAPDH, HPRT and ACTB were used. For all donors the relative expression of TLR3 is increased in the later near- senescent passages. Click here for file
