# Supplementary material for: SORBS2 and TLR3 induce premature senescence in primary human fibroblasts and keratinocytes
Source: BMC Cancer. 2013 Oct 29;13:507. doi: 10.1186/1471-2407-13-507 (PMC3819711; doi:10.1186/1471-2407-13-507)
Supplement: Additional file 1: Table S1 — Primer for real-time PCR. [file 1471-2407-13-507-S1.doc]

| **Table S1 Primer for real-time PCR** | | | |
| --- | --- | --- | --- |
| **Gene** | **F/R** | **Primer** | **T_A_** |
| *SORBS2* | F | TGATAAATGAATAATTCTCTTTGATGCC | 60°C |
|  | R | AATTACCTGGAAGCCAGGTATGAA |  |
| *TLR3* | F | GATTCAAGGTACATCATGCAGT | 54°C |
|  | R | GAAAGGCACCTATCCGTTC |  |
| *CYP4V2* | F | TTGTTTTTAGTGACCCTACATGACAT | 58°C |
|  | R | ATATGAGTAACAATAATTCTGGAGCTGA |  |
| *FBXO18* | F | AGCTGCCCATCACCTATAGCA | 60°C |
|  | R | GCTGTCAGGAACGCCAGG |  |
| *PRKCQ* | F | GAAACGGCCCCATTGC | 58°C |
|  | R | AATTCCCATAAAAACCTATCCAGG |  |
| *IL15RA* | F | CCAAAGCTCTCTGTCAATTACAAGG | 60°C |
|  | R | GGGCTCAGCATCTCTCCCA |  |
| *WDR37* | F | AAACAGTGTTTGGAAGTGGGAAC | 58°C |
|  | R | TGCCCAACAGCATGGCT |  |
| *GATA3* | F | GGGTTTCTTGTTTCTTTTCCATTTT | 60°C |
|  | R | TGCACGCTGGTAGCTCATACA |  |
| *PFKFB3* | F | GAGCCTAAACAATAGAAAGCTGTAGAGA | 60°C |
|  | R | AATTCAGACAAATACACAGAACACAGAGA |  |
| *DIP2C* | F | GTCGTTTGCCGCCTGTG | 60°C |
|  | R | GGGCCAATTCAATTTAGAAGTGC |  |
| *GAPDH* | F | GCGACACCCACTCCTCCACC | 60°C |
|  | R | GAGGTCCACCACCCTGTTGC |  |
| *HPRT* | F | ACGAAGTGTTGGATATAAGC | 52°C |
|  | R | ATAATTTTACTGGCGATGTC |  |
| *ACTB* | F | GGACTTCGAGCAAGAGATGG | 57°C |
|  | R | GAGTGATCTCCTTCTGCATC |  |

T_A_ annealing temperature

F/R forward/reverse primer
