# Supplementary material for: SORBS2 and TLR3 induce premature senescence in primary human fibroblasts and keratinocytes
Source: BMC Cancer. 2013 Oct 29;13:507. doi: 10.1186/1471-2407-13-507 (PMC3819711; doi:10.1186/1471-2407-13-507)
Supplement: Additional file 2: Table S2 — Full-length cDNA-clones and source. [file 1471-2407-13-507-S2.doc]

| **Table S2 Full-length cDNA-clones and source** | | | | | |
| --- | --- | --- | --- | --- | --- |
| **Gene** | **NCBI**  **accession number** | **Source of cDNA clone** | **length of cDNA** | **Vector** | **Reference** |
| *SORBS2-1* | NM_021069.4 ^a^ | pBK-CMV-SORBS2 | 1863 bp | pBK-CMV | Backsch  et al. 2011 |
| *SORBS2-2* | NM_00145671.1 | IRAUp969D0963D | 2825 bp | pOTB7 | * |
| *TLR3* | NM_003265.2 | IRAMp995O012Q | 2964 bp | pCR-BluntII-TOPO | * |
| *CYP4V2* | NM_207352.3 | IRAKp961B19133Q | 1919 bp | pBluescriptR | * |
| *FBXO18* | NM_178150.1 | IRATp970B08111D | 3620 bp | pCMV-SPORT6 | * |
| *IL15RA* | NM_172200.1 | IRAUp969H10107D | 1740 bp | pDNR-LIB | * |
| *WDR37* | NM_014023.3 | DKFZp434F2427Q | 1485 bp | pSPORT1 | * |
| *DIP2C* | NM_014974.2 | IRAKp961K09133Q | 5144 bp | pBluescriptR | * |

^a^  NM_021069.4 but lacking exon 24

* www.imagenes-bio.de
