# Supplementary material for: SORBS2 and TLR3 induce premature senescence in primary human fibroblasts and keratinocytes
Source: BMC Cancer. 2013 Oct 29;13:507. doi: 10.1186/1471-2407-13-507 (PMC3819711; doi:10.1186/1471-2407-13-507)
Supplement: Additional file 3: Table S3 — PCR-terms for long expand template PCR for the different genes. [file 1471-2407-13-507-S3.doc]

| **Table S3**  **PCR-terms for long expand template PCR for the different genes** | | |
| --- | --- | --- |
| **Gene** | **Length** | **T_A_** |
| *SORBS2-1* | 1722 bp | 60 °C |
| *SORBS2-2* | 1935 bp | 60 °C |
| *TLR3* | 2715 bp | 53 °C |
| *CYP4V2* | 1578 bp | 55 °C |
| *FBXO18* | 3131 bp | 53 °C |
| *IL15RA* | 695 bp | 55 °C |
| *WDR37* | 1485 bp | 60 °C |
| *DIP2C* | 4671 bp | 60 °C |

T_A_ = annealing temperature
