# Supplementary material for: SORBS2 and TLR3 induce premature senescence in primary human fibroblasts and keratinocytes
Source: BMC Cancer. 2013 Oct 29;13:507. doi: 10.1186/1471-2407-13-507 (PMC3819711; doi:10.1186/1471-2407-13-507)
Supplement: Additional file 4: Table S4 — Primers with restriction sites for long expand template PCR. [file 1471-2407-13-507-S4.doc]

| **Table S4** **Primers with restriction sites for long expand template PCR** | | | | |
| --- | --- | --- | --- | --- |
| **Gene** | **F/R** | **Primer** | **RS** | **T_A_*** |
| *SORBS2* | F | GCGCGCGCTAGC_ ATGAGTTACTATCAGAGGCCGT | NheI | 63°C |
|  | R | GCGCGCGCGGCCGC_ TCACAGCCTCTTGACGTAG | NotI | 61°C |
| *TLR3* | F | TTTTGCTAGC_ ATGAGACAGACTTTGCCTTGTATCT | NheI | 56°C |
|  | R | TTTTGCGGCCGC_ TTAATGTACAGAGTTTTTGGATCCA | NotI | 56°C |
| *CYP4V2* | F | GCGCGCGGATCC_ ATGGCGGGGCTCTGGC | BamHI | 62°C |
|  | R | GCGCGCGCGGCCGC_ TTAGCGTTCATCTGCATTTCTCCTC | NotI | 62°C |
| *FBXO18* | F | TTTTGCTAGC_ ATGAGACGGTTTAAGCGGAAG | NheI | 57°C |
|  | R | TTTTGCGGCCGC_ TCAGAAGACGAGGAAGAGCAG | NotI | 56°C |
| *IL15RA* | F | GCGCGCGGATCC_ ATGTCCGTGGAACACG | BamHI | 59°C |
|  | R | GCGCGCGCGGCCGC_ TCATAGGTGGTGAGAGCA | NotI | 59°C |
| *WDR37* | F | TTTTGCTAGC_ ATGCCCACAGAAAGCGCAAG | NheI | 62°C |
|  | R | TTTTGCGGCCGC_ TTATTTTTCTTGTAGCAATGCAGGGAT | NotI | 61°C |
| *DIP2C* | F | TTTTTTCGAA_ ATGGCGGACCGCAGCCTG | BstBI | 66°C |
|  | R | TTTTGCGGCCGC_ CTACATGTTGTAGGCCACATAGATGGGG | NotI | 65°C |

RS = restriction site

*T_A_ = annealing temperature without restriction site

F/R = forward/reverse primer
