# Supplementary material for: SORBS2 and TLR3 induce premature senescence in primary human fibroblasts and keratinocytes
Source: BMC Cancer. 2013 Oct 29;13:507. doi: 10.1186/1471-2407-13-507 (PMC3819711; doi:10.1186/1471-2407-13-507)
Supplement: Additional file 5: Table S5 — Primers for sequencing of cDNA-clones. [file 1471-2407-13-507-S5.doc]

| **Table S5**  **Primers for sequencing of cDNA-clones** | | |
| --- | --- | --- |
| **Gene** | **F/R** | **Primer** |
| *SORBS2* | F1 | AGAGCCAGATAACAGTGAATGGAA |
|  | F2 | GTCATCAATTCTTCAGCATGAAAGA |
|  | F3 | AGAGTGGGCATCTTCCCGAT |
| *TLR3* | F1 | TTCCCTGATGAAATGTCTGGAT |
|  | F2 | AAGAGTTTTCTCCAGGGTGTTTTC |
|  | F3 | GTACTTGACCTGGGCCTTAATGA |
|  | F4 | CCTGAGCTGTCAAGCCACTACC |
| *CYP4V2* | F1 | CACTTTCCCGGAGTGCACC |
|  | F2 | GTCCGTGCAGTTTATAGAATGAGTG |
|  | R | TAAAGACCTTTCTCATGATAAAGGCA |
| *FBXO18* | F1 | CAGTAGGGTTTTACAGGTGGGG |
|  | F2 | CCAGGAAGCACTGAGCCAC |
|  | F3 | CCAGGAAGCACTGAGCCAC |
|  | F4 | GACCCGCACCAGCAGATCTAT |
|  | F5 | CAGAGTTGAGTCATTTTCTGAGGAT |
| *IL15RA* | F | AGGCTCCTTCACTCCGGAC |
| *WDR37* | F | GCTGCTGTGACAGCTTATTGC |
|  | R | CCATCAAAGGTCTGTGCTGG |
| *DIP2C* | F1 | AGACCTCCGCCTGCGAAC |
|  | F2 | GGCTCAGACCCACATAGAAAATC |
|  | F3 | CTTGCTTGGAAGCTGTGGAGTTA |
|  | F4 | GAGGCCCACGGATGACAGTA |
|  | F5 | AACAAAACAGCTTTTTCTGGAGG |
|  | F6 | CCCAGACACTCTTGCATATCTCG |
|  | F7 | AAACAAAAGGACCGCTGGG |
| pCDH | F | GTCAGATCGCCTGGAGACG |
|  | R | GCGCTCTGCCCACTGAC |

F = forward primer

R = reverse primer

The annealing temperatures are between 58°C and 60°C.
