# Supplementary material for: SORBS2 and TLR3 induce premature senescence in primary human fibroblasts and keratinocytes
Source: BMC Cancer. 2013 Oct 29;13:507. doi: 10.1186/1471-2407-13-507 (PMC3819711; doi:10.1186/1471-2407-13-507)
Supplement: Additional file 6: Table S6 — Validation with quantitative real-time PCR and evaluation with REST [18] CIN3 vs. CxCa. [file 1471-2407-13-507-S6.doc]

| **Table S6**  **Validation with quantitative real-time PCR and evaluation with REST [18] CIN3 vs. CxCa** | | | | |
| --- | --- | --- | --- | --- |
| **Gene** | **REST**  **CIN3 vs. CxCa (each with 20 samples)** | **p-value** | **REST**  **CIN3 (8) vs. CxCa (9) (samples not on array)** | **p-value** |
| *SORBS2* | -31.709 | <0.001 | -76.847 | <0.001 |
| *TLR3* | -6.437 | <0.001 | -7.53 | <0.001 |
| *CYP4V2* | -6.144 | <0.001 | -4.803 | 0.007 |
| *FBXO18* | -2.445 | <0.001 | -2.222 | 0.0074 |
| *PRKCQ* | -8.048 | <0.001 | -6.076 | 0.0052 |
| *IL15RA* | -2.973 | <0.001 | -2.771 | 0.0276 |
| *WDR37* | -4.676 | <0.001 | -6.078 | <0.001 |
| *GATA3* | -30.061 | <0.001 | -62.647 | <0.001 |
| *PFKFB3* | -1.971 | 0.0174 | -2.034 | 0.087 |
| *DIP2C* | -3.368 | <0.001 | -3.025 | 0.0128 |
