# Supplementary figures and images for: SORBS2 and TLR3 induce premature senescence in primary human fibroblasts and keratinocytes
Source: BMC Cancer. 2013 Oct 29;13:507. doi: 10.1186/1471-2407-13-507 (PMC3819711; doi:10.1186/1471-2407-13-507)

## Slide 1
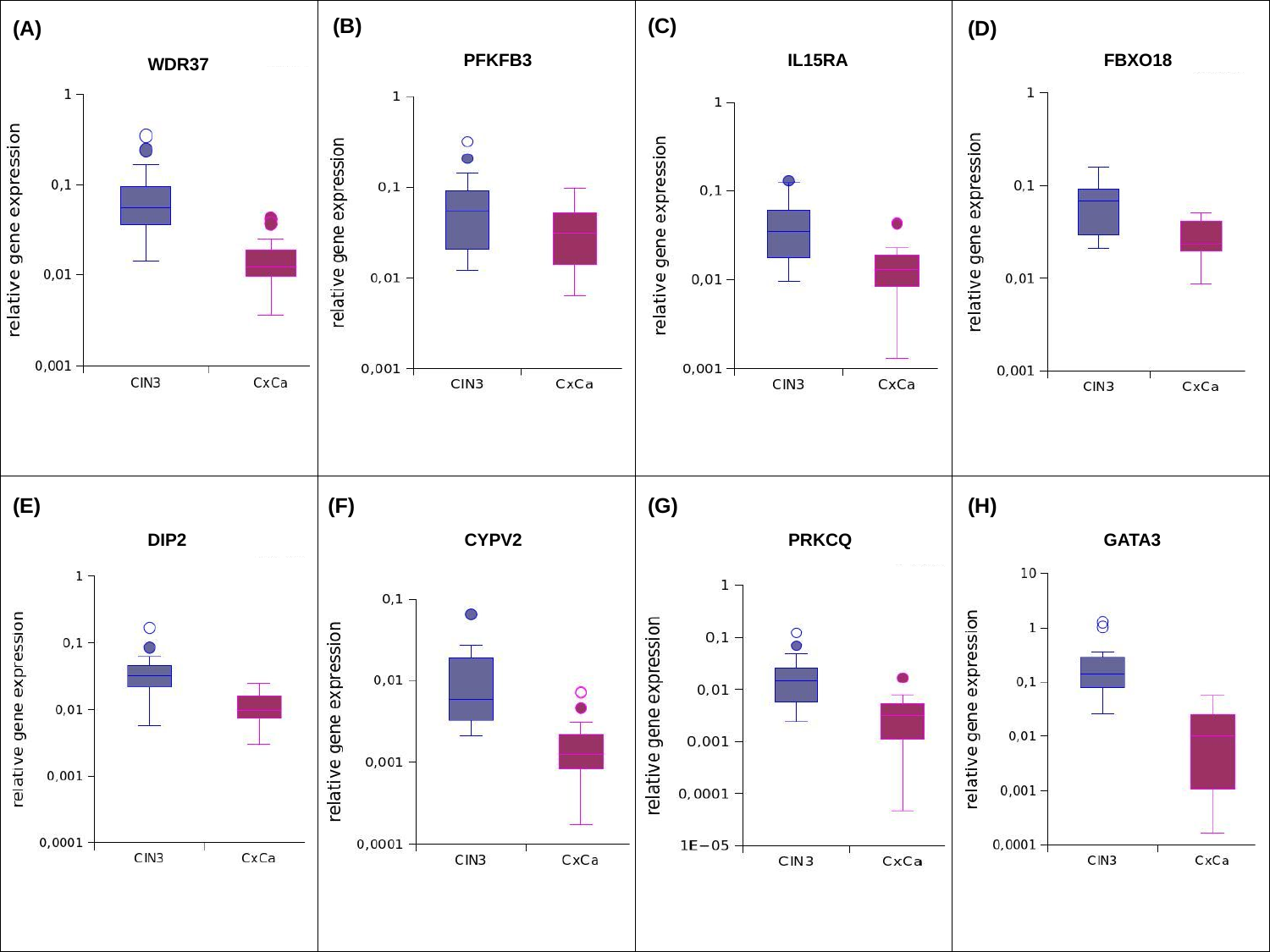

| | | | |
| --- | --- | --- | --- |
| | | | |
(B)
(C)
(A)
(D)
PFKFB3
IL15RA
FBXO18
WDR37
(E)
(F)
(G)
(H)
DIP2
CYPV2
PRKCQ
GATA3

Supplement: Additional file 7: Figure S1 — Expression of candidate genes in CIN3 and cervical carcinomas (CxCa). Relative gene expression of WDR37, PFKFB3, IL15RA, FBXO18, DIP2, CYPV2, PRKCQ, GATA3 was determined by quantitative RT-PCR in CIN3 (n = 20) and CxCa (n = 20). For normalisation the housekeeping genes GAPDH, HPRT and ACTB were used. With exception of PFKFB3 the differences in gene expression were highly significant (<0.001). [file 1471-2407-13-507-S7.ppt]

## Slide 1
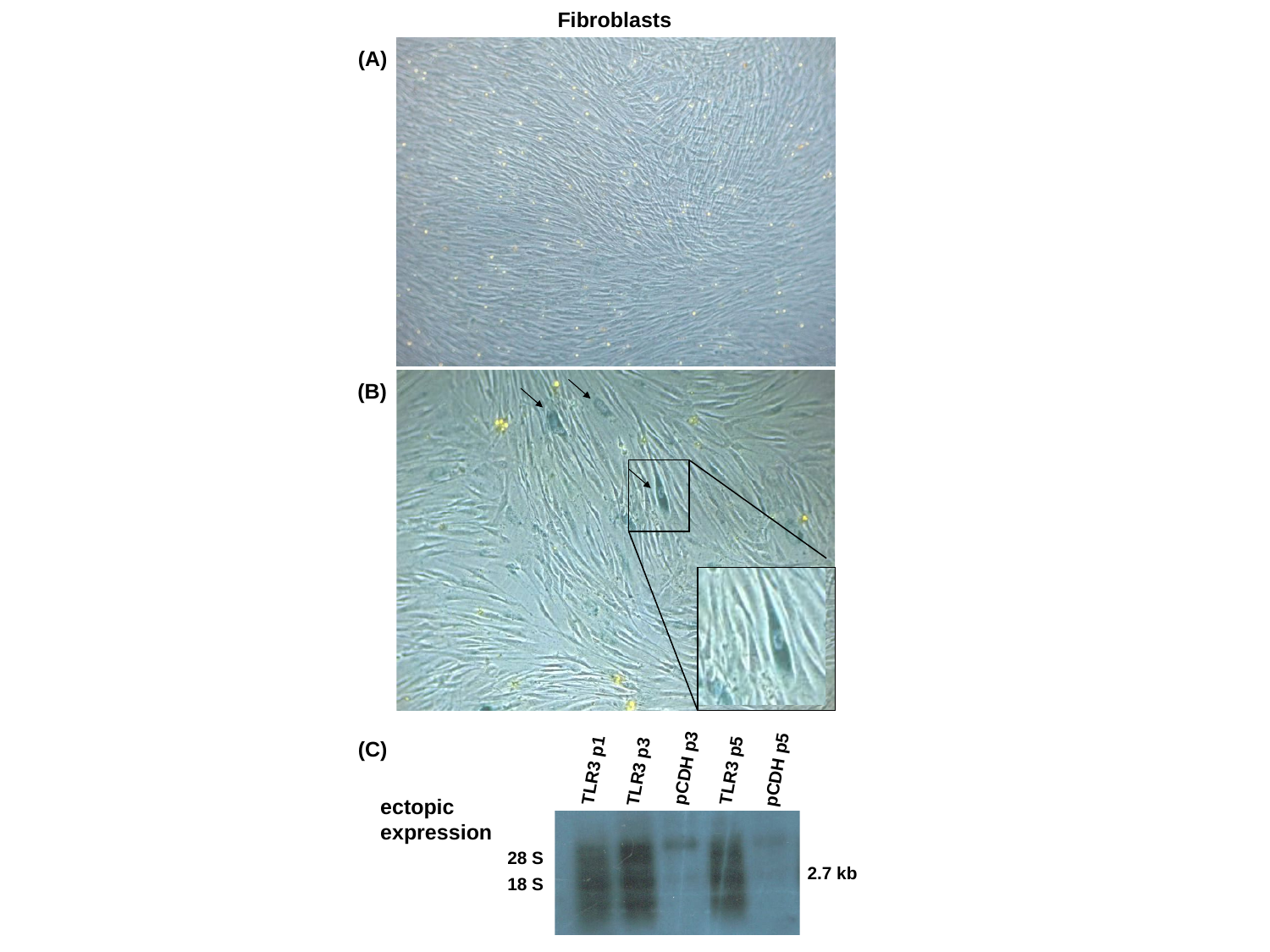

Fibroblasts
(A)
(B)
TLR3 p3
TLR3 p1
pCDH p3
pCDH p5
TLR3 p5
28 S
2.7 kb
18 S
(C)
ectopic
expression

Supplement: Additional file 9: Figure S3 — Ectopic expression of TLR3 in primary fibroblasts Beta-galactosidase staining was performed at different time points after lentiviral transduction. Depicted are cells stained 14 days after transduction of pCDH empty vector (control) and TLR3. Only a few senescent cells are evident in the control (A). An at least 2-fold increase in senescent cells was observed after transduction of TLR3 in primary human fibroblasts (B). The images were captured at 200× magnification. Expression of the transgene in cells at passage 1, 3 and 5 was confirmed by Northern blot (C). [file 1471-2407-13-507-S9.ppt]

## Slide 1
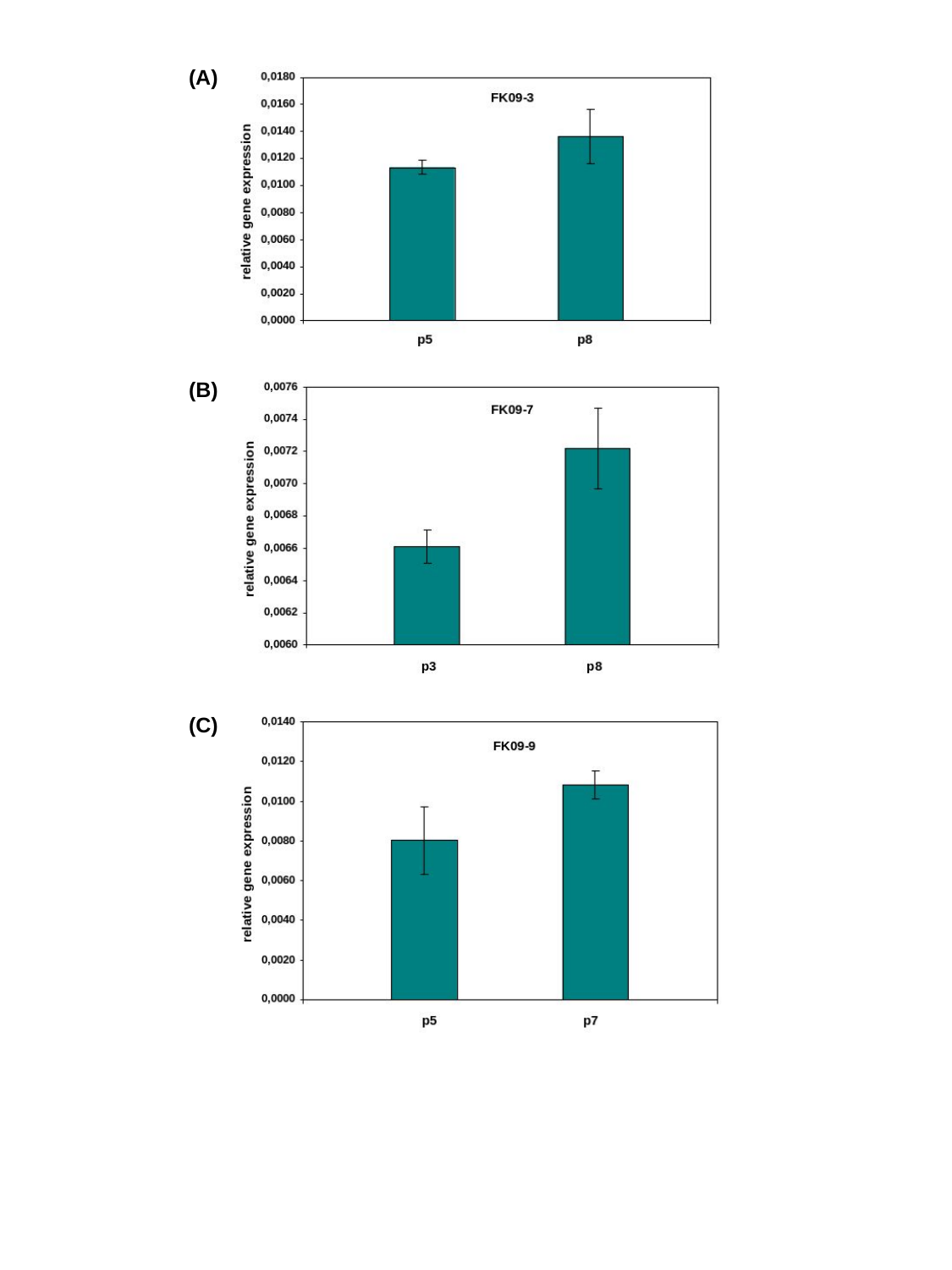

(A)
(B)
(C)

Supplement: Additional file 10: Figure S4 — Endogenous expression of TLR3 during in vitro passaging of primary keratinocytes. Relative gene expression of TLR3 in two different passages from three different donors (FK09-3 (A), FK09-7 (B) and FK09-9 (C)) was determined by quantitative RT-PCR. For normalisation the housekeeping genes GAPDH, HPRT and ACTB were used. For all donors the relative expression of TLR3 is increased in the later near- senescent passages. [file 1471-2407-13-507-S10.ppt]
