# Supplementary material for: SORBS2 and TLR3 induce premature senescence in primary human fibroblasts and keratinocytes
Source: BMC Cancer. 2013 Oct 29;13:507. doi: 10.1186/1471-2407-13-507 (PMC3819711; doi:10.1186/1471-2407-13-507)
Supplement: Additional file 8: Figure S2 — Ectopic expression of SORBS2 in HPV-immortalized cells. Beta-galactosidase staining was performed at different time points after lentiviral transduction. Depicted are cells stained 14 days after transduction of pCDH empty vector (control) and SORBS2-2. Senescence could not be observed in HPKIAp83 and HPKIIp289 (B). Controls were also negative (A). All images were captured at 200× magnification. Expression of the transgene in cells at same passage was confirmed by Western blot (C). [file 1471-2407-13-507-S8.ppt]

## Slide 1
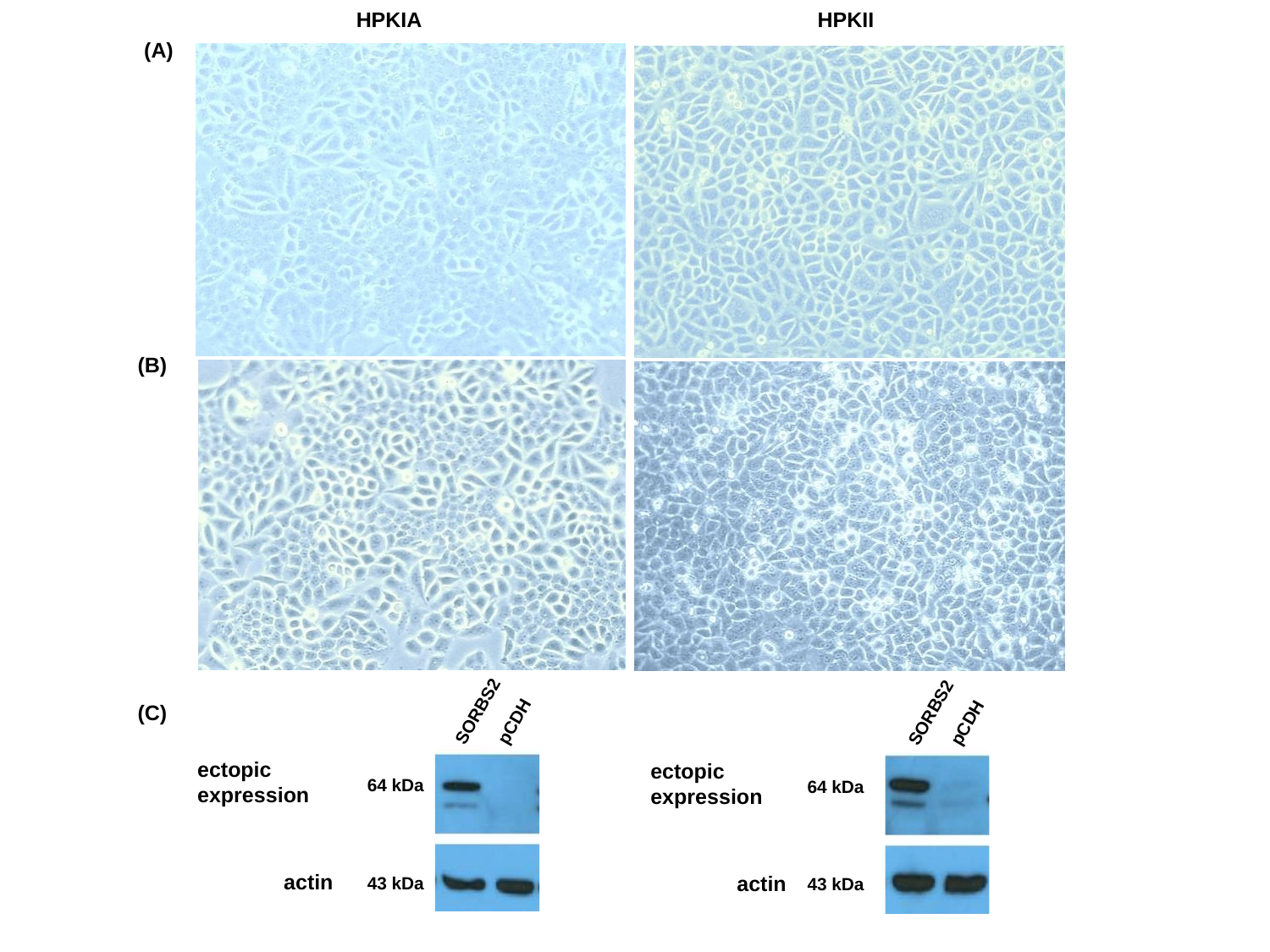

HPKIA
HPKII
(A)
(B)
SORBS2
SORBS2
 pCDH
 pCDH
(C)
ectopic
expression
ectopic
expression
64 kDa
64 kDa
actin
actin
43 kDa
43 kDa
